# TCR diversity – a universal cancer immunotherapy biomarker?

**DOI:** 10.1186/s40425-016-0175-4

**Published:** 2016-11-15

**Authors:** Douglas G. McNeel

**Affiliations:** University of Wisconsin Carbone Cancer Center, 7007 Wisconsin Institutes for Medical Research, 1111 Highland Avenue, Madison, WI 53705 USA

**Keywords:** Sipuleucel-T, Tumor vaccine, Prostate cancer, TCR sequencing, T-cell diversity

## Abstract

Sipuleucel-T was approved as a treatment for men with advanced metastatic, castration-resistant prostate cancer on the basis of improved survival in randomized clinical trials. A major challenge for this therapy, as well as other newer cancer immunotherapy agents, has been to identify markers that can identify patients who benefit from these therapies. In a recent manuscript by Sheikh and colleagues, the investigators evaluated changes in T cell clonality in the peripheral blood and tumors of patients treated with sipuleucel-T using next generation sequencing of T cell receptor Vß CDR3 sequences. Their findings are discussed in the context of this trial and other cancer immunotherapies.

## Editorial

An editorial on: Sheikh, N., J. Cham, L. Zhang, T. DeVries, S. Letarte, J. Pufnock, D. Hamm, J. Trager, and L. Fong. "Clonotypic Diversification of Intratumoral T Cells Following Sipuleucel-T Treatment in Prostate Cancer Subjects." Cancer Res. 2016;76:3711-18.

Dramatic treatment responses with T-cell checkpoint inhibitors for multiple tumor types, and CAR-T cell approaches for B cell malignancies, led the journal *Science* to declare that cancer immunotherapy was the scientific breakthrough of the year in 2013 [1]. Over the last 6 years several immune based treatments have been FDA approved for multiple types of cancer, creating a major paradigm shift in the treatment of many malignancies. One of the first therapies to be approved was sipuleucel-T, an antigen-specific vaccine approved in 2010 for patients with advanced, metastatic, castration-resistant prostate cancer. This treatment was approved on the basis of overall improved survival in patients receiving sipuleucel-T, compared with those receiving placebo, in a phase III randomized trial, with similar improved survival observed in other phase III trials [2].

While studies using sipuleucel-T have shown very little toxicity from treatment [3], a major challenge with the use of sipuleucel-T in clinical practice is that it has been difficult to determine if an individual patient has benefited. That is, few changes in serum PSA or objective radiographic responses are observed after this therapy, changes deemed important by most patients and treating physicians as markers of benefit. Notwithstanding, patients receiving this therapy overall live longer, and appear to have delays in the development of symptomatic disease [4]. This has been observed in animal models of anti-tumor vaccines as well, where tumors may continue to grow, but at a slower pace [5]. This type of treatment effect has also been suggested based on models for another prostate cancer vaccine, rilimogene galvacirepvec/rilimogene glafolivec (Prostvac®, Bavarian Nordic), that is currently being evaluated in a multinational, randomized phase III clinical trial [6]. Retrospective studies have also suggested that patients with lower disease burden may have greater benefit from sipuleucel-T in terms of overall survival than patients with rapidly growing disease [7]. Together, these have suggested that certain patients may benefit more than others from this therapy. Consequently, there has been a need for both predictive and treatment biomarkers to identify patients likely to benefit, and then demonstrate early after therapy whether a patient might have benefitted [8]. Because this treatment is a vaccine targeting a tissue-specific protein, prostatic acid phosphatase (PAP), a logical biomarker evaluation has been whether patients develop systemic immune responses to this target. In fact, the development of antibody responses to PAP was associated with greater benefit in terms of overall survival [9]. Moreover, in other retrospective studies, the development of immune responses to other, non-targeted tumor-associated proteins, a process known as “antigen spread,” was also associated with overall longer survival [10].

Similar needs to identify predictive and treatment biomarkers have occurred following the development of T-cell checkpoint inhibitors such as agents targeting CTLA-4 or PD-1/PD-L1. While objective disease responses do occur in patients treated with these agents, sometimes these responses can occur months after beginning therapy [11]. In the absence of radiographic response, markers to suggest when it is appropriate to continue or abandon these therapies in favor of other treatments are not currently available. However, because these therapies do not act specifically on tumor cells or tumor-specific T cells, but rather affect the global T-cell repertoire, it has not been possible to evaluate immune responses to individual tumor-specific targets such as for a vaccine like sipuleucel-T. Consequently, most biomarker efforts have focused on identifying changes within the tumor microenvironment following these therapies [12]. Earlier studies used immunohistochemistry or flow cytometry to evaluate the cell populations that might be altered within a tumor, evaluating specifically for CD4 and CD8 T cells. In murine studies, the development of CD8 T-cell infiltration has been associated with better anti-tumor responses [13]. Next generation sequencing (NGS) of the complementarity-determining region 3 (CDR3) from rearranged T-cell receptor (TCR) variable beta (Vß) from tumor biopsies has more recently permitted not only the quantitative evaluation of the frequency of T cells within a tissue sample, but also the diversity of T cells, in a fashion irrespective of needing to know what the TCR recognizes. Recent studies using this approach have identified that this approach can be used to evaluate the clonality or diversity of T cells within human tumor specimens [14, 15].

In a recent report by Sheikh, Fong and colleagues in *Cancer Immunology Research,* the authors use NGS of TCR Vß CDR3 sequences to identify changes in T-cell populations both in circulation and within prostate tumors from patients with early stage prostate cancer undergoing treatment with sipuleucel-T [16]. These patients received sipuleucel-T prior to prostatectomy as part of a clinical trial (NCT00715104). While the number of patients evaluated was small, and the authors acknowledge this, importantly they also included evaluation of tissues from a small number of patients who received standard prostatectomy without sipuleucel-T and volunteer blood donors in their analysis to control for natural variation over time. They report that treatment was associated with a decrease in TCR diversity in the peripheral blood over 4 weeks of sipuleucel-T treatment. TCR diversity was higher, however, in prostatectomy specimens from patients treated with sipuleucel-T compared with samples from contemporary patients undergoing prostatectomy without prior treatment. Finally, they identified a higher association of peripheral blood TCR signatures with tumor-associated TCR signatures in patients treated with sipuleucel-T over the course of treatment. They interpret these findings to suggest that sipuleucel-T modulated the tumor-associated immune response, potentially by increasing the antigen-specific immune repertoire, thus narrowing the diversity of T cells in the peripheral circulation. The increased correlation of TCR signatures in the periphery and tumor over the period of treatment suggests that treatment-associated T cells were infiltrating the tumor, and this was further reflected by the increased diversity of T cells at the time of prostatectomy in these individuals compared with patients not receiving sipuleucel-T.

What does this study teach us about sipuleucel-T and prostate cancer? First, the findings of increased TCR diversity in prostate tumors after treatment with sipuleucel-T are consistent with findings from other studies suggesting that active immunotherapies elicit measurable changes within the tumor microenvironment, and increasing TCR diversity might be a “good” thing. Clemente and colleagues, studying large granular lymphocyte leukemia with similar techniques, found a low diversity of TCR species at baseline, suggesting that growing tumors may be characterized by a low diversity, and/or non-functional, infiltrating T cells [17]. Page and colleagues recently reported that increased tumor T-cell frequency was elicited in patients with breast cancer treated with ipilimumab, and that combining this with cyroablation led to increased T-cell numbers and TCR diversity [14]. While the specificity of T cells affected was not determined in the study by Sheikh and colleagues, the changes observed suggests that sipuleucel-T can have an immune-mediated effect within the tumor, supporting the proposed mechanism of action. What is not clear is why TCR diversity would be narrowed in the circulation following sipuleucel-T treatment, proposed to be due to an antigen-specific cell amplification, but then broadened in the tumor microenvironment. In future studies with sipuleucel-T or other vaccines, it would be helpful to know whether the magnitude of T-cell infiltration was affected by treatment, as would be implied by a previous report of this trial [18], but using the TCR sequencing approach. In addition, knowing the specificity of TCR that were amplified would be advantageous, to mechanistically confirm that T cells amplified are specific for the PAP target and/or antigens recognized as those targeted by antigen spread following sipuleucel-T treatment. Unfortunately, because sipuleucel-T has not been extensively studied in this neoadjuvant setting, it is not possible to determine whether any particular individuals “benefited” from treatment, and whether the changes observed were associated with clinical outcome. Thus larger populations with measurable clinical outcomes will be necessary to determine whether changes in TCR diversity might be developed as treatment or predictive biomarkers for sipuleucel-T.

This study is also important by suggesting that changes in T-cell repertoire in the peripheral blood can be measured in response to immunotherapy. This is particularly relevant for prostate cancer, a disease for which early recurrent disease can be detected before the appearance of metastases by radiographic imaging, and for which many metastatic sites are not amenable to biopsy. This may be applicable to other vaccines for prostate cancer or other malignancies, and to other immunotherapy approaches as well. There have been extensive analyses of phenotypic changes in T-cell populations that might be associated with benefit from T-cell checkpoint blockade, however there have been fewer evaluations of the peripheral blood T-cell response in terms of specificity or diversity of response. One study by Postow et al. suggested that a low baseline diversity of TCR frequency was associated with a worse outcome following treatment with ipilimumab [19]. Previous studies by Fong and Robert suggested that increasing TCR diversity after ipilimumab treatment was associated with a better outcome [20, 21]. Studies evaluating whether changes in peripheral blood TCR diversity are associated with better outcome following PD-1/PD-L1 inhibition are underway. There have, however, been extensive analyses of antigen-specific T-cell frequencies following active vaccination. Many of these studies have not identified an association between antigen-specific immunity and clinical outcome. It seems logical that increasing clonality of T cells following vaccination, as in the study of Sheikh and colleagues, could be due to amplification of T cells specific for the target antigen. Alternatively, it is conceivable that oligoclonal expansion of several T-cell species, or CD4 and CD8 species together, might lead to a more favorable outcome than expansion of a single species. Such a finding would be consistent with the recent identification that antigen spread to multiple other cancer-associated proteins was associated with greater benefit following treatment with sipuleucel-T [10]. These will be important studies for the future, and may provide common measures of favorable change in T-cell immunity following different types of immune therapy, whether by vaccines or checkpoint inhibition. Further, the ability to identify changes in the peripheral blood that are associated with changes in the tumor microenvironment could lead to the development of prognostic and treatment biomarkers from peripheral blood samples, an approach more feasible for the majority of tumor types, including prostate cancer, than serial tissue biopsies.

## Abbreviations

CAR-T: Chimeric antigen receptor (expressed in) T cells
CDR3: Complementary-determining region 3
CTLA-4: Cytotoxic T lymphocyte antigen 4
NGS: Next-generation sequencing
PAP: Prostatic acid phosphatase
PD-1: Programmed death receptor 1
TCR: T-cell receptor
